# Function of p21 (Cip1/Waf1/*CDKN1A*) in Migration and Invasion of Cancer and Trophoblastic Cells

**DOI:** 10.3390/cancers11070989

**Published:** 2019-07-15

**Authors:** Nina-Naomi Kreis, Alexandra Friemel, Andreas Ritter, Susanne Roth, Udo Rolle, Frank Louwen, Juping Yuan

**Affiliations:** 1 Department of Gynecology and Obstetrics, School of Medicine, J. W. Goethe-University, D-60590 Frankfurt, Germany; 2 Department of Pediatric Surgery and Pediatric Urology, School of Medicine, J. W. Goethe-University, D-60590 Frankfurt, Germany

**Keywords:** p21, *CDKN1A*, trophoblastic cell lines, invasion, ERK3/MAPK6, MMP2, TIMP2

## Abstract

Tumor progression and pregnancy have several features in common. Tumor cells and placental trophoblasts share many signaling pathways involved in migration and invasion. Preeclampsia, associated with impaired differentiation and migration of trophoblastic cells, is an unpredictable and unpreventable disease leading to maternal and perinatal mortality and morbidity. Like in tumor cells, most pathways, in which p21 is involved, are deregulated in trophoblasts of preeclamptic placentas. The aim of the present study was to enlighten p21’s role in tumorigenic choriocarcinoma and trophoblastic cell lines. We show that knockdown of p21 induces defects in chromosome movement during mitosis, though hardly affecting proliferation and cell cycle distribution. Moreover, suppression of p21 compromises the migration and invasion capability of various trophoblastic and cancer cell lines mediated by, at least partially, a reduction of the extracellular signal-regulated kinase 3, identified using transcriptome-wide profiling, real-time PCR, and Western blot. Further analyses show that downregulation of p21 is associated with reduced matrix metalloproteinase 2 and tissue inhibitor of metalloproteinases 2. This work evinces that p21 is involved in chromosome movement during mitosis as well as in the motility and invasion capacity of trophoblastic and cancer cell lines.

## 1. Introduction

The hallmarks of cancer include sustaining proliferative signaling, evading growth suppressors, resisting cell death, enabling replicative immortality, inducing angiogenesis, and activating invasion and metastasis [1,2]. Aberrant cell migration and invasion contribute to metastasis responsible for over 90% of cancer-associated deaths [3]. Tumor progression and placenta development have many common features including proliferation and invasion [4]. Malignant tumors and placental trophoblasts use similar biochemical mediators to facilitate invasion, therefore, trophoblasts have been termed ”pseudomalignant tissue” [5]. Invasion of extravillous trophoblasts (EVT) into the maternal decidua is essential for placental embedment and fetal development [4]. Failures in this process have been implicated in a wide spectrum of pregnancy disorders like preeclampsia (PE). The pathophysiology of PE is associated with profound cellular dysfunctions including deregulated proliferation, enhanced apoptosis, defective differentiation with poor invasion of trophoblastic cells, and oxidative stress [6,7,8]. With a global prevalence of about 8%, it is one of the leading causes of maternal and perinatal mortality and morbidity [7]. Despite decades of research, the detailed pathophysiology and molecular mechanisms of PE are still poorly understood.

The balance and control over cell cycle progression is ensured by distinct cell cycle mediators. An important cell cycle regulator is p21 (Cip1/Waf1), the founding member of cyclin-dependent kinase (Cdk) inhibitors and encoded by the gene *CDKN1A*. p21 is involved in various vital cellular processes like cell cycle regulation including mitosis, DNA repair, apoptosis, differentiation, cytoskeletal dynamics, cell migration, gene transcription, reprogramming of induced pluripotent stem cells, aging, and the onset of senescence [9,10]. Interestingly, p21 functions not only as tumor suppressor, but also as oncogene with dual behavior in different cellular processes with frequently opposite cellular responses, partially depending on its subcellular localization, interaction partners, and its phosphorylation status [9,11]. The oncogenic part has so far been attributed to its cytoplasmic localization [9]. This notion is, however, challenged by the recent observation that p21 can also exhibit oncogenic functions through replication stress triggered by its sustained expression and its nuclear accumulation in a p53-null background [12]. Thus, p21, described as “linchpin” [13] of cell fate, is tightly involved in balancing different pathways in molecular oncology as well as placentology including cell cycle regulation, differentiation, senescence, and invasion. While p21 is one of the most fascinating research topics in oncology, there are limited studies on cell cycle regulators in placentology [14]. It has nevertheless been shown that p21 is expressed in distinct cell types of primary trophoblasts like EVTs throughout normal pregnancy linking p21 to proliferation, differentiation, and invasion [15,16,17]. Though its expression in trophoblasts of preeclamptic placentas is contradicting, p21 has been considered as a marker for cell cycle arrest, senescence, and apoptosis [18,19,20,21].

In the present work, we have characterized the roles of p21 in diverse trophoblast-derived choriocarcinoma and invasive trophoblastic cell lines from first trimester placenta, partially corroborated with cervical carcinoma cell line HeLa. We show here that the reduction of p21 with two different small interfering RNA (siRNAs) impaired the motility and invasion capacity of various cell lines without influencing their proliferation pattern.

## 2. Results

### 2.1. Expression of p21 in Trophoblastic and Choriocarcinoma Cell Lines

**Figure 1:**
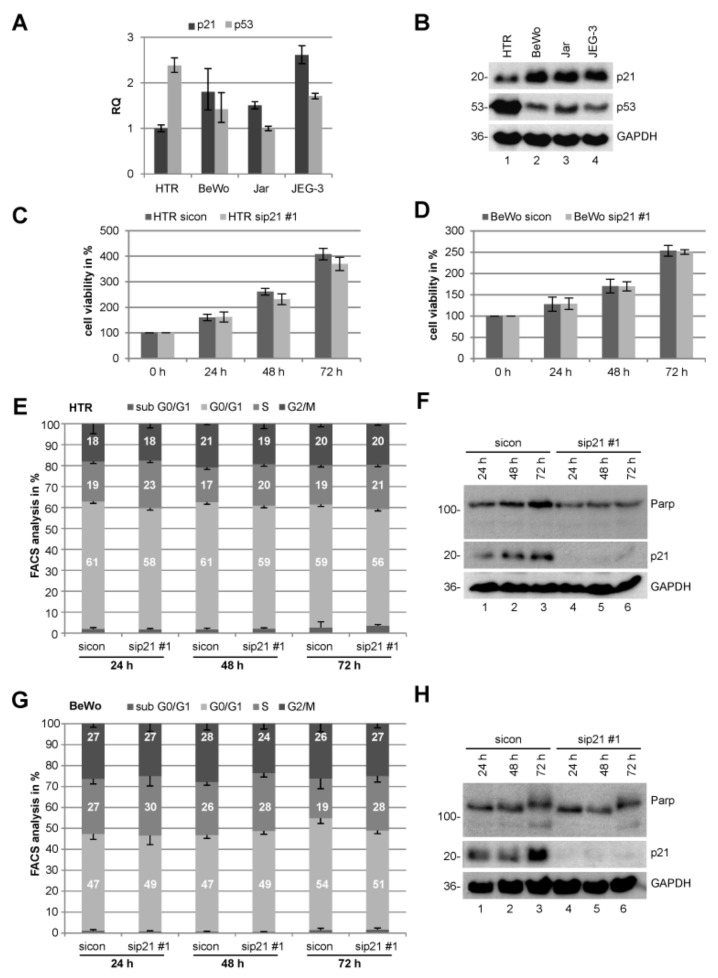
Knockdown of p21 barely impacts proliferation and cell cycle distribution of choriocarcinoma or trophoblastic cells. (**A**) Real-time PCR of *CDKN1A* (p21) and *TP53* (p53). The results are presented as RQ with minimum and maximum range. RQ: relative quantification of gene expression by setting p21 of HTR cells as 1 or p53 of Jar cells, respectively. (**B**) Western blot analysis of HTR, BeWo, Jar, and JEG-3 cells. Glyceraldehyde-3-phosphate dehydrogenase (GAPDH) served as loading control. (**C**) HTR cells were treated with control small interferingRNA (siRNA) (sicon) or siRNA against p21 (sip21 #1) for 0, 24, 48, and 72 h. Cell viability was measured via CellTiter-Blue^®^ assay (Promega, Mannheim, Germany). The results are presented as mean ± standard error of the mean (SEM) (*n* = 2, each experiment in triplicates) and statistically analyzed compared to sicon. All differences were not significant. (**D**) Cell viability assay of BeWo cells treated as in (C). (**E**) Fluorescence-activated cell scanning (FACS) measurements of HTR cells for cell cycle distribution. The results are presented as mean ± SEM from three independent experiments. (**F**) Cellular extracts from HTR cells were prepared for Western blot analyses with indicated antibodies. GAPDH served as loading control. (**G**) FACS measurements of BeWo cells as in (**E**). (**H**) Cellular extracts from BeWo cells were prepared for Western blot analyses with indicated antibodies. GAPDH served as loading control.

In order to gain insight into p21’s messenger RNA (mRNA) and protein expression, we used four well-characterized trophoblastic cell lines: BeWo, JEG-3, and Jar cells, all three derived from choriocarcinoma, and HTR-8/SVneo, referred to hereafter as HTR, an immortalized first trimester trophoblast cell line [22]. Interestingly, the expression of p21 and its master regulator p53 behaves reciprocally at the mRNA and protein level (Figure 1A,B). p53 has been reported to be wild type without mutations in villous trophoblasts and choriocarcinoma [6]. The first trimester-derived HTR cell line displayed a strong p53 and a lesser p21 mRNA as well as protein signal, whereas all three choriocarcinoma cell lines showed a strong p21 and a lesser p53 level (Figure 1A,B), implicating a different control of p21’s expression between normal trophoblasts and malignant trophoblast-derived cell lines. For instance, the p21 transcriptional repressor *c-MYC* (myelocytomatosis oncogene cellular homolog) [23] is highly expressed in HTR cells and cytotrophoblasts of early gestational weeks [24,25], which might cause the strong reduction of p21 despite high levels of p53. Besides, p21 is exceedingly regulated by a myriad of different transcriptional p53-independent controllers and it is induced in differentiated cells [26], which could explain the observed levels in choriocarcinoma cells.

### 2.2. Knockdown of p21 Does Not Change the Proliferation Capacity Neither Cell Cycle Distribution

Acquired deregulated cell proliferation and cell cycle control are hallmarks of cancer cells as well as preeclamptic trophoblasts. To address the role in proliferation, p21 was knocked down in HTR and BeWo cells with siRNA against the 3’ untranslated region (UTR) of p21 (referred to as sip21 #1) followed by cell viability assays up to 72 h. There was no notable difference in proliferation in cells treated with sip21 #1 compared to control siRNA (sicon) in both cell lines (Figure 1C,D). To study cell cycle distribution of these cells, fluorescence-activated cell scanning (FACS) analyses were performed. Both HTR and BeWo cells showed hardly any alterations in their cell cycle distribution (Figure 1E,G). The cells were also harvested for the examination of apoptosis induction via Western blot analyses using antibody against poly (adenosine diphosphate (ADP)-ribose) polymerase (PARP) and its cleaving product. No remarkable difference was observed between cells depleted of p21 and control cells (Figure 1F,H, upper panel). Comparable results were obtained from Jar and JEG-3 cells (Figure S1). Taken together, normal trophoblastic as well as malignant choriocarcinoma cell lines transiently depleted of p21 with siRNA show no notable differences in their proliferation capacity, cell cycle distribution, or apoptotic induction in 2D culture systems.

### 2.3. Suppression of p21 Affects Chromosome Segregation of Choriocarcinoma and Trophoblastic Cell Lines

**Figure 2:**
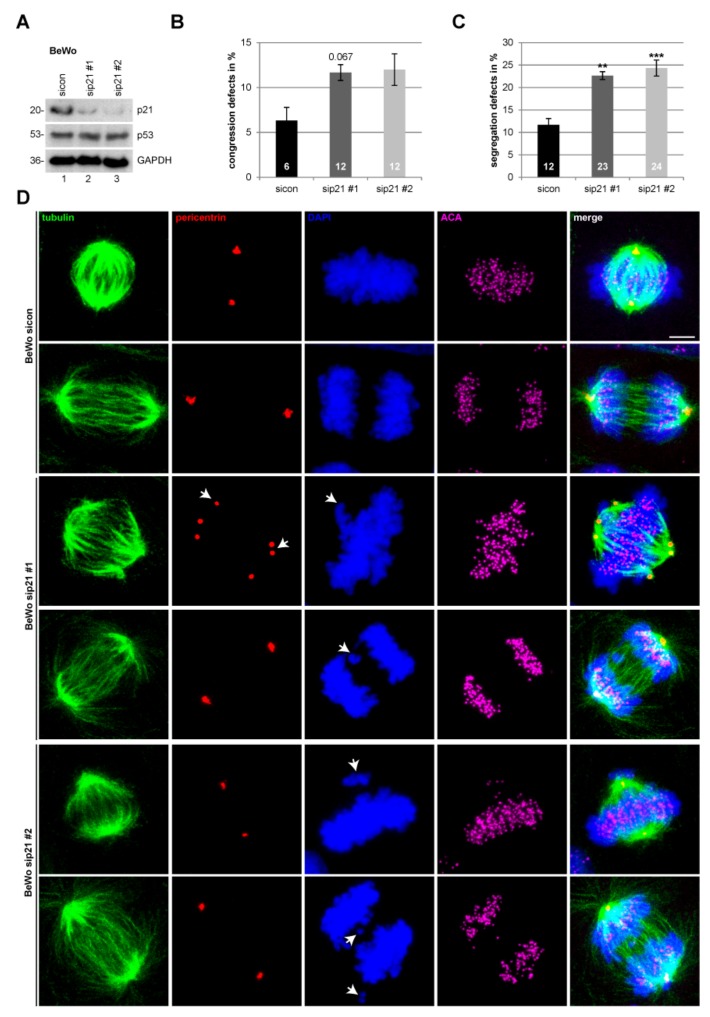
Suppression of p21 affects chromosome segregation in BeWo cells. (**A**) Western blot control for small interfering RNA (siRNA) transfection efficiency. BeWo cells were treated with scrambled siRNA (sicon) or siRNA against the untranslated region (UTR) of p21 (sip21 #1) or mixed siRNAs against the coding region of p21 (sip21 #2) for 48 h. (**B**) Quantification of defects in chromosome congression in metaphase cells treated as in (A) (*n* = 3, 100 metaphase cells per experiment and condition). The results are presented as mean ± standard error of the mean (SEM) and statistically analyzed. (**C**) Quantification of defects in chromosome segregation in anaphase cells treated as in (A) (*n* = 3, 100 anaphase cells per experiment and condition). The results are presented as mean ± SEM and statistically analyzed. ** *p* < 0.01; *** *p* < 0.001. (**D**) Representative images of confocal laser scanning microscopy are shown. Cells were stained for tubulin, pericentrin, anti-centromere antibody (ACA), and DNA. Scale bar: 7.5 µm. Arrow: Indicating either chromosome congression/segregation defect (4’,6-diamidino-2-phenylindole dihydrochloride (DAPI) staining) or failure of centrosome integrity (pericentrin staining).

Besides its versatile functions, p21 is also important for mitotic progression and chromosome integrity [9]. Studies with various cancer lines including colon cancer HCT116 p21 wild-type and p21-knockout (p53 wild-type), as well as cervical carcinoma HeLa (p53 inactive) and osteosarcoma Saos-2 (p53 deficient) cell lines treated with sip21 #1, demonstrated that depletion of p21 causes mitotic defects independent of its p53 status [27]. To study if similar effects could also be observed in choriocarcinoma and trophoblastic cell lines, BeWo cells were treated with control siRNA and two different siRNAs targeting p21 (Figure 2A). sip21 #1 is directed against the UTR region, whereas sip21 #2 is a pool of different siRNAs against the coding region of p21. The treated cells were fixed and stained for microtubule marker tubulin, centrosome marker pericentrin, and centromere marker ACA (anti-centromere antibody) and DNA (Figure 2D). Metaphase and anaphase cells were examined via a microscope. Interestingly, compared to control cells, knockdown of p21 resulted in twice as many segregation errors in BeWo cells (Figure 2C). These cells also showed increased congression failures, though the difference is not significant (Figure 2B). The same experiment was performed with SGHPL-4 cells, which are also derived from first trimester placenta [28]. Like BeWo cells, depletion of p21 induced a significant increase in chromosome segregation errors in SGHPL-4 cells (Figure S2).

Since p21 deficiency causes centriole overduplication with aberrant centrosome numbers in murine myeloblasts [29] and in colorectal cancer cells upon ionizing radiation [30], we analyzed the morphology and number of centrosomes in metaphase cells of choriocarcinoma cell line BeWo and trophoblastic cell line SGHPL-4. Abnormal centrosomes were subdivided into multipolarity and centrosome clustering at a pole. The analysis showed no significant difference between control siRNA and sip21 treated BeWo (Figure 2D, panel 3 and Figure S3A) or SGHPL-4 cells (Figure S2D, panel 5 and S3B). Of notice, though not influenced by p21, placental-derived cells display centrosome clustering, as reported in BeWo and Jar cells [31].

### 2.4. Motility and Invasion Capacity are Lowered in Cells Treated with siRNA Against p21

**Figure 3:**
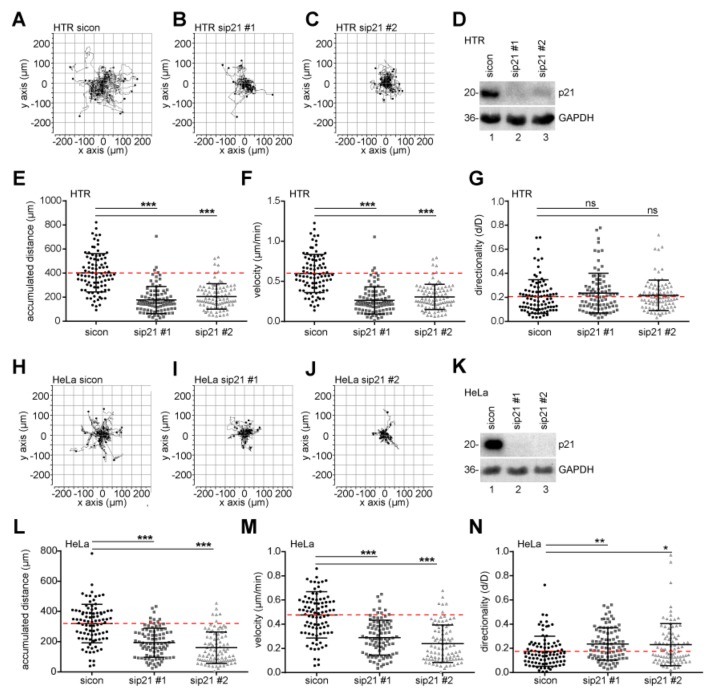
p21 is required for cell motility. Single-cell tracking using time-lapse microscopy was performed with treated HTR cells. (**A**–**C**) Representative trajectories of individual cells (*n* = 30) treated with sicon (A), sip21 #1 (**B**), or sip21 #2 (C) are shown. (**D**) Control Western blot analysis shows the efficient knockdown of endogenous p21 in HTR cells. Glyceraldehyde-3-phosphate dehydrogenase (GAPDH) served as loading control. (**E**–**G**) Accumulated distance (E), velocity (F), and directionality (**G**) of these cells were analyzed and statistically evaluated from three independent experiments. The results are shown as scatter plots. *** *p* < 0.001. (**H**–**J**) Representative trajectories are shown for individual HeLa cells (*n* = 30) with sicon (**H**), sip21 #1 (**I**), and sip21 #2 (J). (**K**) Control Western blot analysis of HeLa cells. GAPDH served as loading control. (**L**–**N**) Accumulated distance (L), velocity (M), and directionality (N) were evaluated. The data are derived from three independent experiments and shown as scatter plots. * *p* < 0.05, ** *p* < 0.01, *** *p* < 0.001.

Trophoblastic cells are highly invasive and motile by nature, sharing similar signaling pathways with cancer cells and representing an attractive model for studying cell migration and invasion processes. Using time-lapse microscopy, single HTR cells, treated with control siRNA or two different siRNAs targeting p21 (Figure 3D), were tracked and their migratory parameters were evaluated (Figure 3A–C). We observed a strong reduction in motility, particular in the accumulated distance (Figure 3E) and the velocity (Figure 3F) of p21-depleted cells, whereas the directionality was scarcely changed (Figure 3G). Similar results were obtained with cervical carcinoma cell line HeLa upon treatment with two different siRNAs targeting p21 (Figure 3H–N).

**Figure 4:**
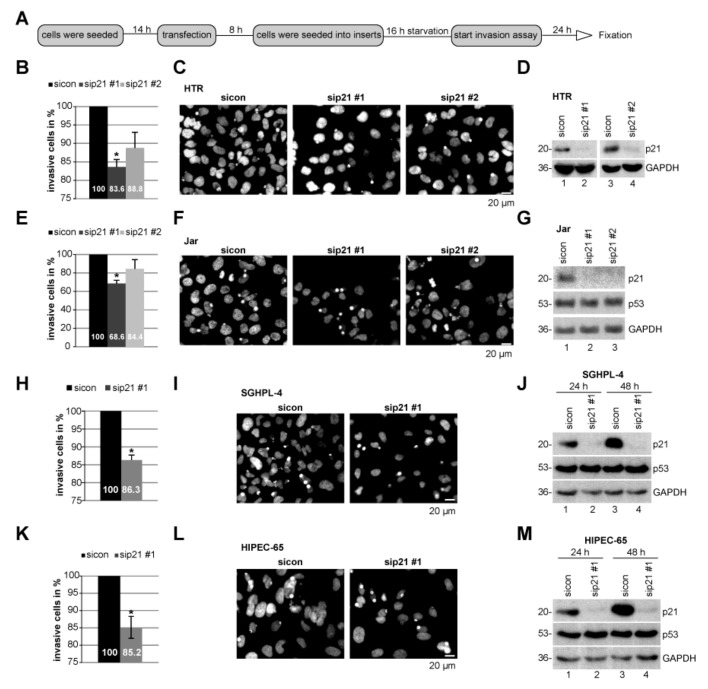
The invasion capacity is impaired in cells with reduced p21 levels. (**A**) Schedule of invasion assay. Different cell lines were treated with control small interfering RNA (siRNA) (sicon) or two distinct siRNA against p21 (sip21 #1 or #2). (**B**) Quantification of invaded HTR cells. The total number of invaded cells in the control group was assigned as 100%. (**C**) Representatives of invaded HTR cells are shown. Scale bar: 20 μm. (**D**) Control Western blot analysis showing the efficient knockdown of endogenous p21 in HTR cells. Glyceraldehyde-3-phosphate dehydrogenase (GAPDH) served as loading control. (**E**) Quantification of invaded Jar cells. (**F**) Representatives of invaded Jar cells are shown. Scale bar: 20 μm. (**G**) Control Western blot analysis of Jar cells. GAPDH served as loading control. (**H**) Quantification of invaded SGHPL-4 cells. (**I**) Representatives of invaded SGHPL-4 cells are shown. Scale bar: 20 μm. (**J**) Control Western blot analysis of SGHPL-4 cells. GAPDH served as loading control. (**K**) Quantification of invaded HIPEC-65 cells. (**L**) Representatives of invaded HIPEC-65 cells. Scale bar: 20 μm. (**M**) Control Western blot analysis of HIPEC-65 cells. GAPDH served as loading control. The results from each cell line are presented as mean ± standard error of the mean (SEM) from three independent experiments. * *p* < 0.05.

To underline these observations, invasion assays of cells passing a matrigel in a transwell system were performed [32]. Compared to HTR cells transfected with control siRNA, HTR cells depleted of p21 with sip21 #1 displayed a moderately reduction in the number of invaded cells by 16% and those transfected with sip21 #2 by 11%, respectively (Figure 4B–D). Interestingly, choriocarcinoma Jar cells depleted of p21 showed a decrease in invasion up to 31% with sip21 #1 and 16% with sip21 #2 (Figure 4E–G). Like HTR cells, suppression of p21 with sip21 #1 decreased this ability by 14% and 15% in trophoblastic SGHPL-4 and HIPEC-65 [33], respectively (Figure 4H–M).

### 2.5. ERK3 Expression is Reduced Upon p21 Silencing

**Figure 5:**
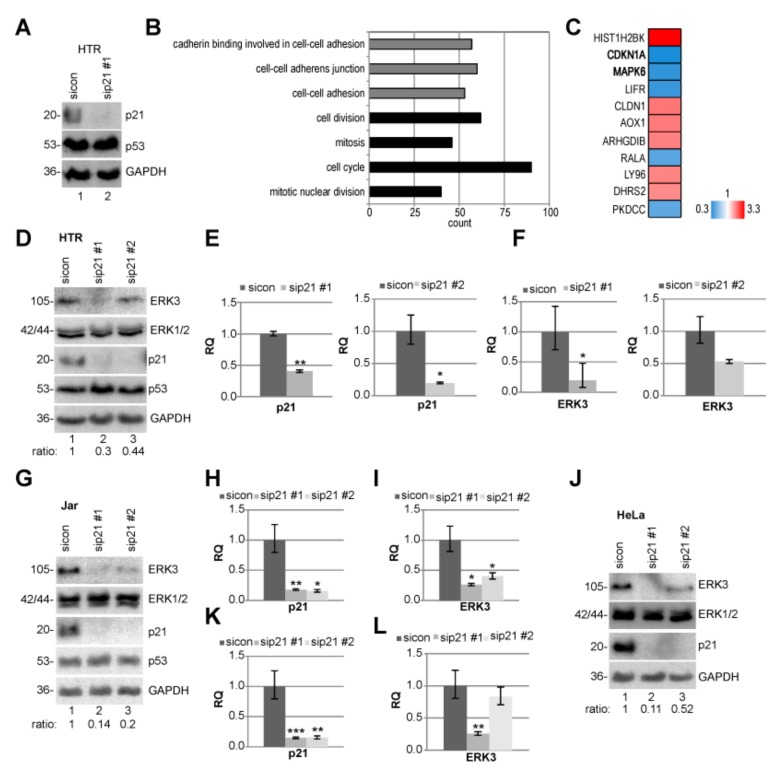
Extracellular signal-regulated kinase 3 (ERK3) is reduced in p21-depleted cell lines. (**A**–**C**) Microarray analysis. HTR cells were treated with sicon or sip21 #1 for 48 h and the RNA from three independent experiments was extracted. (**A**) Western blot control of HTR cells with glyceraldehyde-3-phosphate dehydrogenase (GAPDH) as loading control. (**B**) The effects of sip21 #1 on different pathways were determined by gene ontology enrichment analysis using Database for Annotation, Visualization and Integrated Discovery (DAVID) [37]. (**C**) Heatmap of the most differently expressed genes. Gene expression was analyzed using HumanHT-12 v4 beadchip array (Illumina, San Diego, CA, USA). Genes with a *p*-value < 0.01, and a fold change greater than 2 (red color code) and below 0.5 (blue color code), respectively, are included. (**D**) Western Blot of HTR cells treated with control siRNA (sicon) or two distinct siRNA against p21 (sip21 #1 or #2) for 48 h. Ratio of ERK3/GAPDH is shown. GAPDH served as loading control. (**E**,**F**) The gene levels of p21 (E) and ERK3 (F) were measured. (**G**) Western blot analyses of Jar cells treated as in (D). (**H**,**I**) The gene levels of p21 (H) and ERK3 (I). (**J**) Western blot analyses of HeLa cells treated as in (D). (**K**,**L**) The gene levels of p21 (K) and ERK3 (L). The mRNA data are based on three experiments and presented as RQ with minimum and maximum range. RQ: relative quantification of gene expression. * *p* < 0.05, ** *p* < 0.01, *** *p* < 0.001.

To look deeply at possible mechanisms by which p21 depletion causes defects in motility and invasion, we performed RNA microarray analysis using a HumanHT-12 v4 beadchip array (Figure 5A–C). Compared to HTR cells treated with sicon, we analyzed differently expressed genes in p21-depleted HTR cells. The evaluation of various pathways affected by p21 knockdown was determined by gene ontology enrichment analysis from genes with a *p*-value smaller than 0.05 with functional annotation clustering using Database for Annotation, Visualization and Integrated Discovery (DAVID) [32]. These analyses identified that genes of cell–cell adhesion, cell cycle, and mitosis are enriched (Figure 5B and Table S1). Genes with a *p*-value smaller than 0.01, and a fold change greater than 2 (red color code) and below 0.5 (blue color code), respectively, are displayed in a heatmap (Figure 5C and Table S2). The extracellular signal-regulated kinase 3 (ERK3), encoded by the gene *MAKP6*, is among the top 3 of altered genes and strongly reduced upon p21-depletion (hit number 2, *CDKN1A*). ERK3 is a distantly related member of the mitogen-activated protein kinase (MAPK) superfamily [34], which is known to be involved in cell differentiation [35] and migration [36]. To validate the sequencing results, HTR cells were transfected with sicon and two different siRNAs targeting p21, and the protein and mRNA level of ERK3 were measured. The ERK3 protein was indeed reduced upon p21-depletion with a more demonstrative result from sip21 #1; whereas the level of ERK1/2 was not altered (Figure 5D). Like the protein level, its mRNA was also strongly decreased upon the treatment with sip21 #1 (Figure 5E–F). These results were further corroborated in Jar as well as HeLa cells (Figure 5G–L).

### 2.6. MMP2 and TIMP2 mRNA Are Reduced Upon p21 Depletion

**Figure 6:**
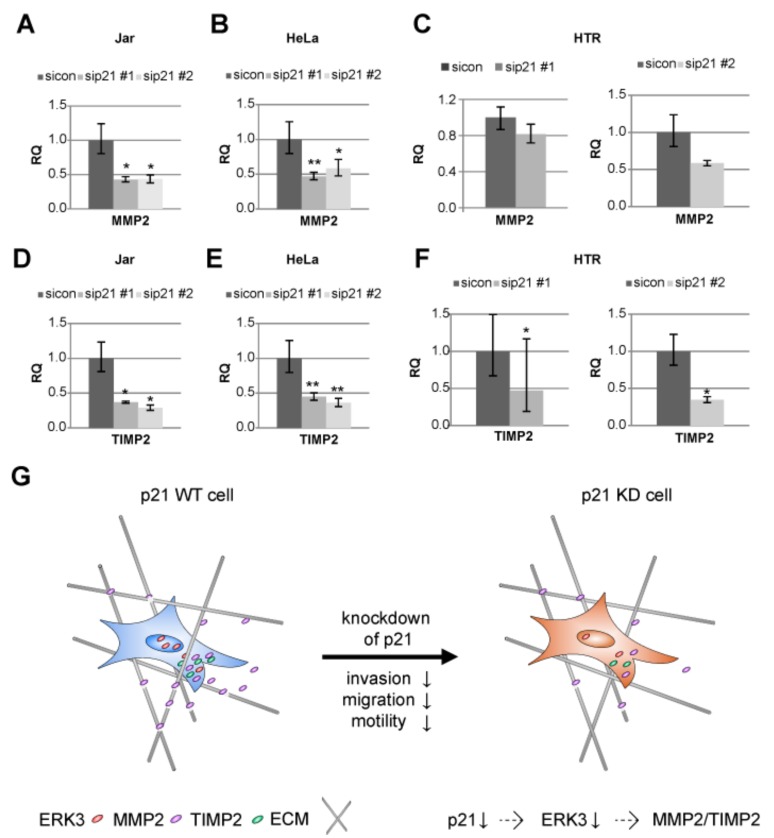
Matrix metalloproteinase 2 (MMP2) and tissue inhibitor of metalloproteinase 2 (TIMP2) are reduced upon p21-silencing. The gene levels of MMP2 (**A**–**C**) and TIMP2 (**D**–**F**) were evaluated from Jar (A and D), HeLa (B and E), and HTR (C and F) cell lines. The data are based on three independent experiments and presented as RQ with minimum and maximum range and statistically analyzed. RQ: relative quantification of the gene expression. * *p* < 0.05, ** *p* < 0.01. (**G**) Schematic illustration of the proposed working model.

Cancer cell and trophoblast invasion are closely linked to the expression of matrix metalloproteinases (MMPs), which are able to degrade the extracellular matrix (ECM) [38]. MMPs like MMP2 are regarded as key enzymes in the invasion process of trophoblastic cells [5,39,40]. MMPs are also known to be influenced by ERK3 expression [41]. Therefore, we asked if the mRNA of MMP2 is altered in p21-depleted cells with reduced ERK3 level. A reduction in MMP2 mRNA expression was indeed observed in Jar cells (Figure 6A). Further support was obtained from cervical carcinoma cell line HeLa (Figure 6B) and trophoblastic HTR cells (Figure 6C).

Moreover, the activation of MMP2 is dependent on tissue inhibitor of metalloproteinases 2 (TIMP2) and membrane-anchored proteinase MMP14/membrane-type (MT)-MMP [42]. As TIMP2 is required for the activation of the latent pro-form of MMP2 [42], we analyzed the mRNA expression of TIMP2 in three different cell lines upon depletion of p21 with two different siRNAs. In fact, a strong reduction of TIMP2 mRNA was detected in the choriocarcinoma cell line Jar, in the cervical carcinoma cell line HeLa and the invasive trophoblastic cell line HTR (Figure 6D–F).

In sum, the data suggest that the depletion of p21 has a negative impact on cell motility and invasion mediated by, at least partially, the ERK3/MMP2/TIMP2 pathway.

## 3. Discussion

With the present work we have studied the roles of p21 in various placental choriocarcinoma and invasive trophoblastic cell lines derived from first trimester placenta. Though p21 affects hardly cell cycle distribution and proliferation, its depletion induces defects in chromosome alignment and segregation. Moreover, suppression of p21 impairs the motility and invasion capacity of diverse trophoblastic cell lines. Attenuated invasion/motility capacity is related to reduced ERK3 levels further influencing the expression of MMP2 and TIMP2, which are involved in the degradation of the extracellular matrix and important for the invasion process.

Like in cancer cells [9,27], p21 is important for successful chromosome segregation and the absence of p21 results in mitotic defects in placenta-derived cell lines. Unlike cancer cells, where depletion of p21 resulted in impaired proliferation rates and enhanced apoptosis, especially upon usage of mitotic drugs [9,27,43], they were not affected in choriocarcinoma and trophoblastic cells depleted of p21. These data suggest that the regulation of proliferation and apoptosis induction in trophoblastic cells differ from that in cancer cells and that p21 is less required for these processes under normal circumstances. Interestingly, p21 as target of Notch-1 is involved in cell cycle arrest of JEG-3 and SGHPL-5 cells (a variant of SGHPL-4) upon treatment with recombinant human glycosylated cysteine-rich angiogenic protein 61(CCN1) and nephroblastoma overexpressed (NOV/CCN3) proteins, important extracellular matrix signaling proteins [44,45].

The role of p21 in cell motility is multifaceted and depends on the cellular context. For instance, nuclear p21 functioning as transcription factor/co-factor is essential for transforming growth factor β (TGFβ)-mediated breast cancer cell migration and invasion, whereas its gene silencing blocked the tumor invasion in a mammary fat pad xenograft mouse model and various breast cancer cell lines, without alterations in cell growth and proliferation [46]. In this study, high expression of p21 correlates with poor survival of breast cancer patients promoting migration/invasion [46]. In support of this, another study with a tumor mouse model showed that invasion is accompanied by an upregulation of p21 pointing to a ”reciprocal switching between proliferation and invasion” mediated by p21 [47]. p21 knockout mice showed dramatic suppression of metastasis, which was independent of tumor growth and rescuable by re-expression of p21 [47]. Cytoplasmic p21 leads to an inhibition of the Ras homolog gene family, member A (RhoA)/ Rho-associated, coiled-coil-containing protein kinase (ROCK)/ LIM domain kinase (LIMK)-pathway and loss of actin stress fibers favoring cell motility in murine embryonic fibroblasts [48], whereas others report that the complex of cytoplasmic p21 and p53 suppresses invasion due to favoring apoptotic induction [49]. Moreover, p21 together with p16 promote tumor growth in mice by enhancing the chemotaxis of monocytic myeloid-derived suppressor cells as observed with double-knockout mice [50]. In contrast, the *CDKN1A*^SUPER^ mouse harboring a third *CDKN1A* allele has a tumor-suppressive phenotype under genotoxic stress [51]. Trophoblasts have to escape from the cell cycle before the invasion process takes place, which includes the activation of p21 [45]. The role of p21 in migration/invasion is thus dependent on environmental and cellular contexts, which is not unexpected, considering the reciprocal impact between versatile factors and p21. In trophoblastic cells, the depletion of p21 demonstrates a decrease in motility and invasion capacity without influencing cell proliferation or apoptosis induction.

We report here that reduced motility and invasion capability of trophoblastic cells is, at least partially, mediated by reduced ERK3. ERK3 is a distantly related member of the MAP kinase family [34] with roles in proliferation, cell cycle progression, and differentiation [35]. ERK3 can activate its first identified substrate MAP kinase-activated protein kinase 5 (MK5 also called PRAK) affecting actin remodeling and cell migration [52]. ERK3, like p21, is a substrate of Cdk1/cyclin B1 in mitosis [53,54]. ERK3 is stabilized upon Cdk1 phosphorylation [53] and activated through phosphorylation by group I p21-activated kinases [55]. ERK3 promotes cancer cell migration/invasion and tumor metastasis, and its expression is up-regulated in multiple cancers like breast, lung, and head and neck [56]. Similar to p21, cytoplasmic ERK3 is involved in enhanced migration and invasion in HeLa and A549 cells [57]. Moreover, ERK3 regulates cell morphology and promotes breast cancer cell migration and spreading [36]. The direct correlation between ERK3 and p21 has hardly been investigated so far and our data suggest that p21 is related to the transcriptional expression of ERK3 in trophoblastic cells as well as in cancer cells, possibly in an indirect manner. 

In addition to ERK3 reduction, suppression of p21 is related to a decrease in MMP2 gene expression upon p21 depletion. ERK3 stimulates lung cancer cell invasiveness both in vitro and in vivo by phosphorylating the steroid receptor coactivator 3 oncoprotein, which causes an upregulation of different MMP genes including MMP2 [41]. The knockdown of ERK3 caused an inhibition of lung cancer cell invasion but without affecting actin polymerization [41]. The reduction of ERK3 and MMP2, as observed in our study upon p21-depletion, could contribute to the diminished cell motility and invasion capacity of diverse cell lines. 

Moreover, we observed a decline in TIMP2 expression upon p21 depletion. While TIMP2 is originally described as member of a family responsible for the inhibition of MMPs, it is also required for the efficient activation of pro-MMP2 as shown by the generation of TIMP2-knockout mice [42] leading to contradictory results concerning TIMP2 expression and its predicted function in human cancer [58]. Its overexpression as well as its downregulation is associated with poor prognosis and distant metastasis [58,59]. Interestingly, it has been reported that a decrease in TIMP2 expression causes an inhibition of trophoblast invasion [60].

The microarray analysis revealed even more interesting candidate genes including leukemia inhibitory factor receptor (*LIFR*), which is involved in invasion of choriocarcinoma JEG-3 [53] and trophoblastic HTR cells [54,55]. LIFR was shown to contribute to an upregulation of MMP14 expression [55], also important for MMP2 activation. Interestingly, LIFR is reduced in HTR cells upon depletion of p21. Moreover, the gene *ARHGDIB* encodes the protein Rho guanosine diphosphate (GDP)-dissociation inhibitor 2. Its overexpression inhibited cell migration in HTR cells [56]. *ARHGDIB* is enriched upon p21-depletion. Further investigations are required to study the relationship of p21 with these candidates and their functions in migration and invasion in trophoblastic cells.

Taken together, p21 reduction leads to attenuated cell motility and invasion capability of different cell lines, possibly through a reduction of ERK3 further resulting in a decline in MMP2 and TIMP2 gene levels (Figure 6G).

## 4. Materials and Methods 

### 4.1. Cell Culture and siRNA Transfection

BeWo (Sigma-Aldrich, Taufkirchen, Germany), Jar, JEG-3, and HeLa cells were cultured as instructed (ATCC, Wesel, Germany). HTR-8/SVneo cell line (referred to as HTR), SGHPL-4 cell line, and the HIPEC-65 cell line were kindly provided by Prof. Graham [22], Prof. Whitley [28], and Prof. Fournier [33], respectively. All cell lines were cultured as instructed by provider. siRNA targeting p21 (sense: ACACCUCCUCAUGUACAUA and antisense: UAUGUACAUGAGGAGGUGU; designated as sip21 #1) was manufactured by Sigma-Aldrich (Taufkirchen, Germany). A different siRNA against p21 (referred to as sip21 #2), containing a mixed pool of siRNAs, was from Santa Cruz (Heidelberg, Germany; sc-29427). Control siRNA was obtained from Qiagen (Hilden, Germany; 1027281). siRNAs (20–30 nM) were transiently transfected with Oligofectamine^TM^ (Thermo Fisher Scientific, Dreieich, Germany), as reported [54]. 

### 4.2. Western Blot Analysis

Cellular lysates were prepared using RIPA buffer (50 mM Tris pH 8.0, 150 mM NaCl, 1% NP-40, 0.5% Na-desoxycholate, 0.1% sodium dodecyl sulfate (SDS), 1 mM NaF, phosphatase, and protease inhibitor cocktail tablets (Roche, Mannheim, Germany)). Western blot analysis was performed as previously described [61]. The following antibodies were used for Western blot analysis: Mouse monoclonal antibody against glyceraldehyde-3-phosphate dehydrogenase (GAPDH) (GTX627408) from GeneTex (BIOZOL, Eiching, Germany), p53 (sc-126) from Santa Cruz Biotechnology (Heidelberg, Germany), rabbit polyclonal antibodies against ERK3/MAPK6 (#4067) and PARP (#9542) from Cell Signaling (Frankfurt, Germany), rabbit polyclonal antibody against ERK1/2 (#06-182) from Merck Millipore (Darmstadt, Germany), and rabbit monoclonal antibody against p21 (#2947) from Cell Signaling. Raw data of all Western blots are supplied as Figures S4–S6. Quantification of Western blot analysis was performed with ImageJ 1.48v software (National Institutes of Health, Bethesda, MD, USA). All densitometry measurements of all Western blot bands are shown in Table S3. 

### 4.3. RNA Extraction, Real-Time PCR and Data Analysis

Total RNAs were extracted with EXTRACTME Total RNA Kit without DNase digestion according to manual instructions (7Bioscience GmbH, Hartheim, Germany). Reverse transcription was performed using Go Script Reverse Transcription Mix as instructed (Promega, Mannheim, Germany). The primers and probes for GAPDH (Hs_02786624), p21 (Hs_00355782), ERK3 (Hs0083126), MMP2 (Hs_01548727), and TIMP2 (Hs00234278) were from Applied Biosystems (Darmstadt, Germany). Real-time PCR was performed with a StepOnePlus Real-time PCR System, and the data were analyzed via StepOne Software v2.3 (Applied Biosystems, Darmstadt, Germany). The final results are presented as relative quantification (RQ) as previously described [31].

### 4.4. Cell Viability, Cell Cycle Measurements, and Invasion Assay 

Cell proliferation assays were performed using CellTiter-Blue^®^ Cell Viability Assay (Promega, Mannheim, Germany) as described [43]. Cell cycle was analyzed using a FACSCalibur^TM^ (BD Biosciences, Heidelberg, Germany), as shown [61]. For invasion assay, cells were seeded in 24-well transwell matrigel chambers according to the manufacturer’s instructions (Corning, New York, USA) and as previously reported [62]. Briefly, cells (75,000 cells of HTR, SGHPL-4, HIPEC-65; and 55,000 cells of Jar) were seeded into the upper chamber of the transwell in 500 μL serum-free medium and the lower chamber was filled with 750 μL serum-free medium. After 16 h, the medium of the lower chamber was discarded and the invasion assay was started by adding medium containing 10% fetal bovine serum for further 24 h. Cells were fixed with ethanol and stained with 4’,6-diamidino-2-phenylindole dihydrochloride (DAPI). Invaded cells were imaged with an AxioObserver.Z1 microscope (Zeiss, Göttingen, Germany) and analyzed with ImageJ 1.50i (National Institutes of Health, Bethesda, MD, USA) and cell counter plugin. 

### 4.5. Cell Motility Evaluation via Time-Lapse Microscopy

Cells were seeded into 24-well plates with a low confluency and were imaged at 5-minute time intervals for 13 hours. Time-lapse imaging was performed with an AxioObserver.Z1 microscope (Zeiss, Göttingen, Germany), imaged with an AxioCam MRc camera (Zeiss, Göttingen, Germany) equipped with an environmental chamber to maintain proper environmental conditions (37 °C, 5% CO_2_). The time-lapse movies were analyzed by using ImageJ 1.49i software (National Institutes of Health, Bethesda, MD, USA) with the manual tracking plugin, and Chemotaxis and Migration Tool (Ibidi GmbH, Gräfelfing, Germany). Tracks were derived from raw data points and were plotted in GraphPad Prism 7 (GraphPad software Inc., San Diego, CA, USA). The accumulated distance was calculated by using the raw data points by the Chemotaxis and Migration Tool. Thirty random cells per experiment were analyzed and the experiments were repeated independently three times. The patterns of motility were evaluated and migration velocity and directionality was calculated as descripted previously [32].

### 4.6. Immunofluorescence Staining

For indirect immunofluorescence, cells were seeded on Nunc^TM^ Lab-Tek^TM^ II CC2^TM^ chamber slides from Thermo Fisher Scientific (Schwerte, Germany). For indirect immunofluorescence staining, cells were fixed with 4% paraformaldehyde containing 0.2% Triton X-100 for 15 min at room temperature as described [54]. The following primary antibodies were used: rabbit polyclonal antibody against pericentrin (ab4448, Abcam, Cambridge, UK), mouse monoclonal antibody against α-tubulin (Sigma-Aldrich, Taufkirchen,, Germany, T5168), and human immune serum against centromere (anti-centromere antibody HCT-0100, ACA, ImmunoVision, Springdale, USA). Secondary antibodies were obtained from Jackson Immunoresearch (Cambridgeshire, UK). DNA was stained using DAPI (4’,6-diamidino-2-phenylindole dihydrochloride) (Roche, Mannheim, Germany). Slides were examined using an AxioObserver (Zeiss, Göttingen, Germany). Z1 microscope and images were taken using an AxioCam MRm camera (Zeiss, Göttingen, Germany). The slides were further examined by confocal laser scanning microscopy (CLSM) using Z-stack images with a HCXPI APO CS 63.0 × 1.4 oil objective (Leica CTR 6500, Heidelberg, Germany). A series of Z-stack images were captured at 0.5 μm intervals for overlays (superimposing individual images from confocal Z-sections). 

### 4.7. Microarray Analysis

HTR cells were treated with control siRNA or siRNA targeting p21#1 for 48 h. Cells from three independent experiments were harvested and the total RNA was isolated using RNeasy Kits (Qiagen, Hilden, Germany). The expression was assessed using Human HT-12 v4 Beadchip (Illumina, San Diego, CA, USA), a direct hybridization whole-gene expression array. Therefore, the Expression Profiling Service from the German Cancer Research Center (DKFZ Microarray Core Facility, Heidelberg, Germany) was used. Most significant genes with a *p*-value (Student’s t-test) less than 0.05 were selected and gene ontology enrichment analysis was carried out using DAVID, and the most enriched gene clusters were shown [37].

### 4.8. Statistical Analysis

Student’s *t*-test (two tailed and paired or homoscedastic) was used to evaluate the significant difference between diverse groups for cell titer, invasion assay, and real-time-PCR. The statistical evaluation of the single cell tracking assay was performed by using an unpaired Mann–Whitney *U* test (two tailed). Difference was considered as statistically significant when *p* < 0.05.

## 5. Conclusions

Tumor progression and placenta development have many features in common. Like in tumor cells, p21 is required for chromosome integrity during mitosis of trophoblastic cells. More importantly, this study shows that suppression of p21 compromises the capability of trophoblastic cells in migration and invasion. This is, at least partially, mediated by the ERK3/MMP2/TIMP2 network in trophoblasts. Further work is required to decipher the impact of p21 on the development of PE, which will also deliver insights into cancer and stem cell research.

## Supplementary Materials

The following are available online at https://www.mdpi.com/2072-6694/11/7/989/s1, Figure S1: Depletion of p21 hardly changes the cell cycle distribution of Jar and JEG-3 cells, Figure S2: Knockdown of p21 affects chromosome segregation in SGHPL-4 cells, Figure S3: Knockdown of p21 barely affects centrosome integrity, Figure S4: Raw data of Western blots from Figure 1 to Figure 3, Figure S5: Raw data of Western blots from Figure 4 to Figure 5G, Figure S6: Raw data of Western blots from Figure 5J to Figure S2, Table S1: Data from whole gene-expression array with a p-value smaller than 0.05, Table S2: Most significant genes for the heatmap, Table S3: Densitometry measurements of all Western blot bands.
