# Supplementary material for: Function of p21 (Cip1/Waf1/CDKN1A) in Migration and Invasion of Cancer and Trophoblastic Cells
Source: Cancers (Basel). 2019 Jul 15;11(7):989. doi: 10.3390/cancers11070989 (PMC6678555; doi:10.3390/cancers11070989)
Supplement: Supplementary file 1 [file cancers-11-00989-s001.pdf]

Supplementary Materials

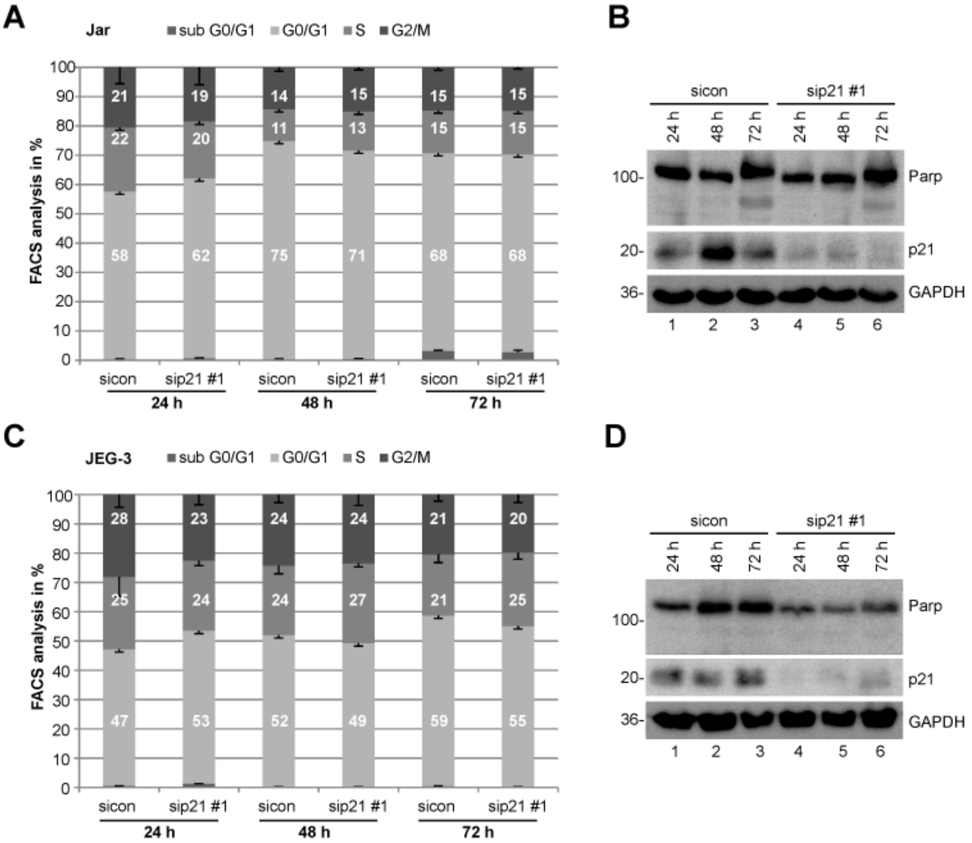

Figure S1. Depletion of p21 hardly changes the cell cycle distribution of Jar and JEG-3 cells. (**A**) Fluorescence-activated cell scanning (FACS) measurements of Jar cells for cell cycle distribution. The results are from three independent experiments and presented as mean ± standard error of the mean (SEM). (**B**) Cellular extracts from Jar cells were prepared for Western blot analyses with indicated antibodies. Glyceraldehyde-3-phosphate dehydrogenase (GAPDH) served as loading control. (**C**) FACS measurements of JEG-3 cells for cell cycle distribution. The results are from three independent experiments and presented as mean ± SEM. (**D**) Cellular extracts from JEG-3 cells were prepared for Western blot analyses with indicated antibodies. GAPDH served as loading control.

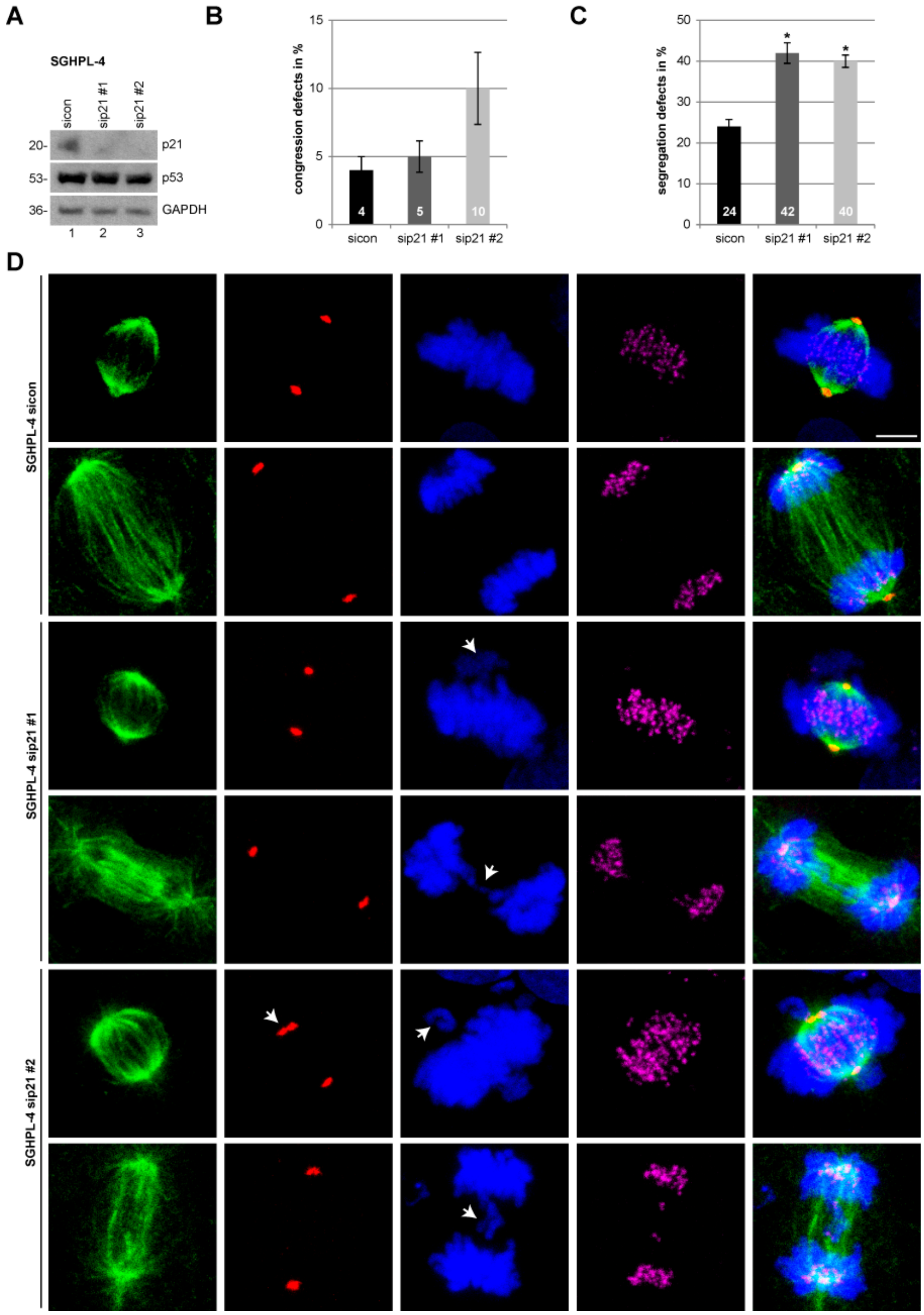

Figure S2. Knockdown of p21 affects chromosome segregation in SGHPL-4 cells. (**A**) Western blot control for small interfering RNA (siRNA) transfection efficiency. SGHPL-4 cells were treated with scrambled siRNA (sicon) or siRNA against the untranslated region (UTR) of p21 (sip21 #1) or a mixed siRNAs against the coding region of p21 (sip21 #2) for 48 h. (**B**) Quantification of defects in chromosome congression in metaphase cells treated as in (**A**) (n = 3, 100 metaphase cells per experiment and condition). The results are based on three independent experiments, presented as mean ± standard error of the mean (SEM) and statistically analyzed. (**C**) Quantification of defects in chromosome segregation in anaphase cells treated as in (A) (n = 3, 100 anaphase cells per experiment and condition). The results are derived from three independent experiments, presented as mean ± SEM and statistically analyzed. * $p < 0.05$. (**D**) Representative images of confocal laser scanning microscopy are shown. Cells were stained for tubulin, pericentrin, anti-centromere antibody (ACA) and DNA. Scale bar: 5 μm. Arrow: indicating either chromosome congression/segregation defect (4′,6-diamidino-2-phenylindole dihydrochloride (DAPI) staining) or failure of centrosome integrity (pericentrin staining).

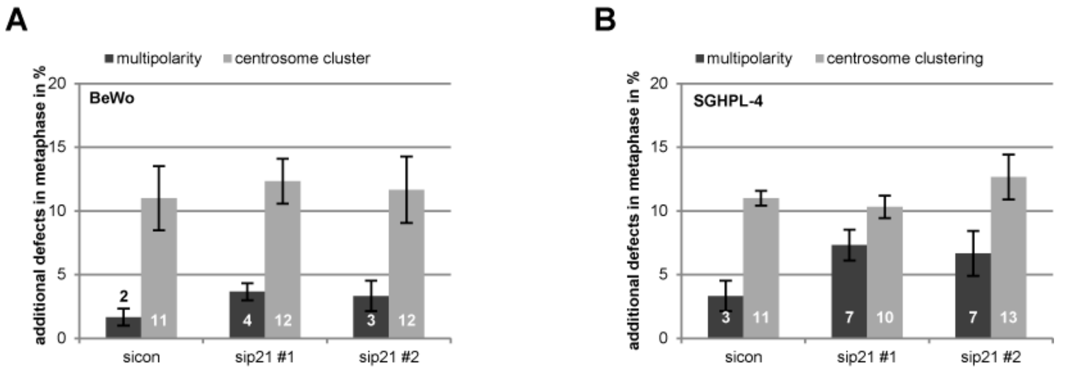

Figure S3. Knockdown of p21 barely affects centrosome integrity. (**A**) Quantification of multipolarity and the abundance of centrosome clustering from BeWo cells treated as in Figure 2 (n = 3, 100 metaphase cells per experiment and condition). The results are from three independent experiments and presented as mean ± standard error of the mean (SEM). (**B**) Quantification of multipolarity and the abundance of centrosome clustering from SGHPL-4 cells treated as in Figure 2 (n = 3, 100 metaphase cells per experiment and condition). The results are from three independent experiments and presented as mean ± SEM.

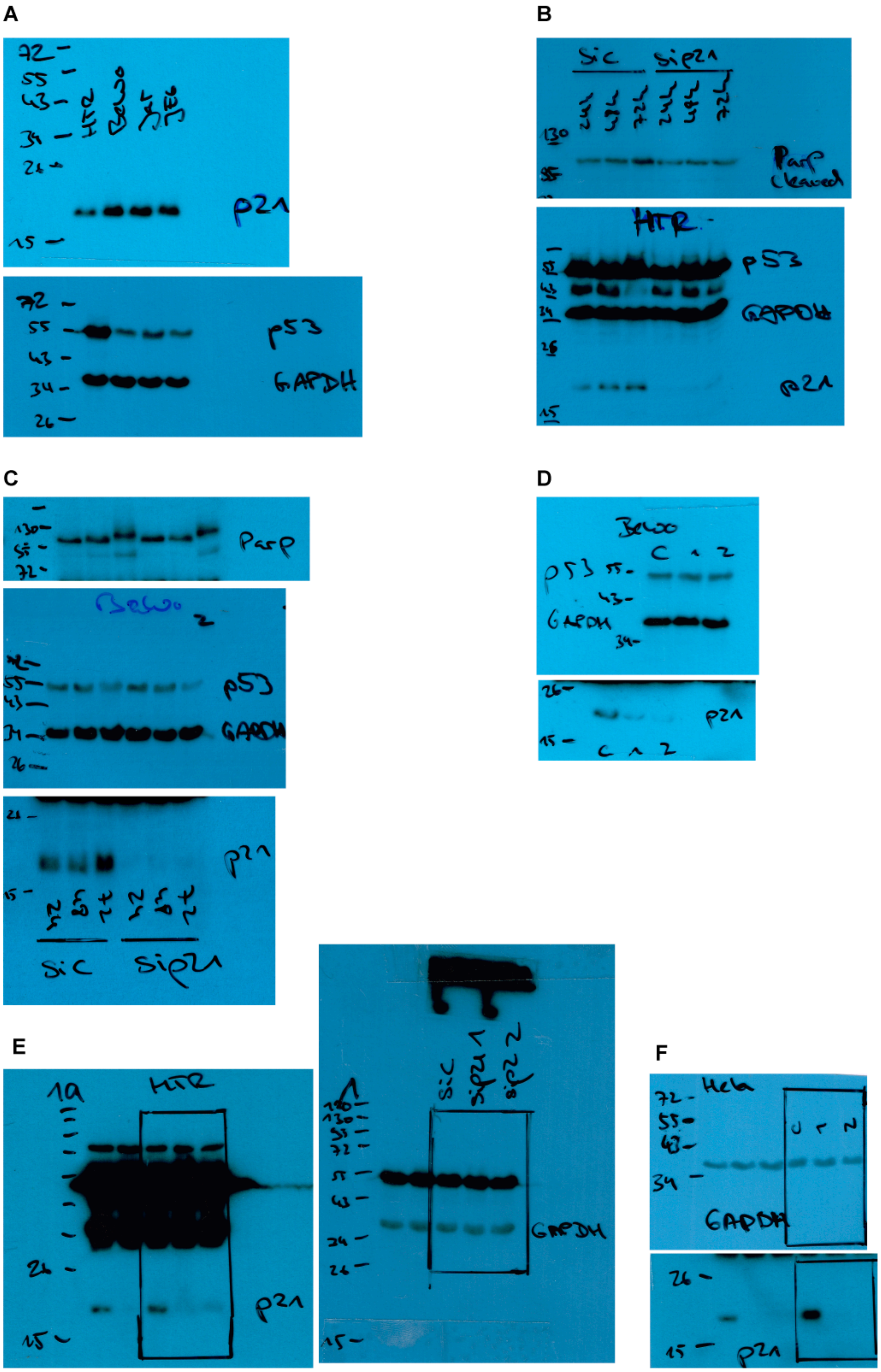

Figure S4. Raw data of Western blots from Figure 1 to Figure 3. (**A**) Raw data from Figure 1B. (**B**) Raw data from Figure 1F. (**C**) Raw data from Figure 1H. (**D**) Raw data from Figure 2A. (**E**) Raw data from Figure 3D. (**F**) Raw data from Figure 3K.

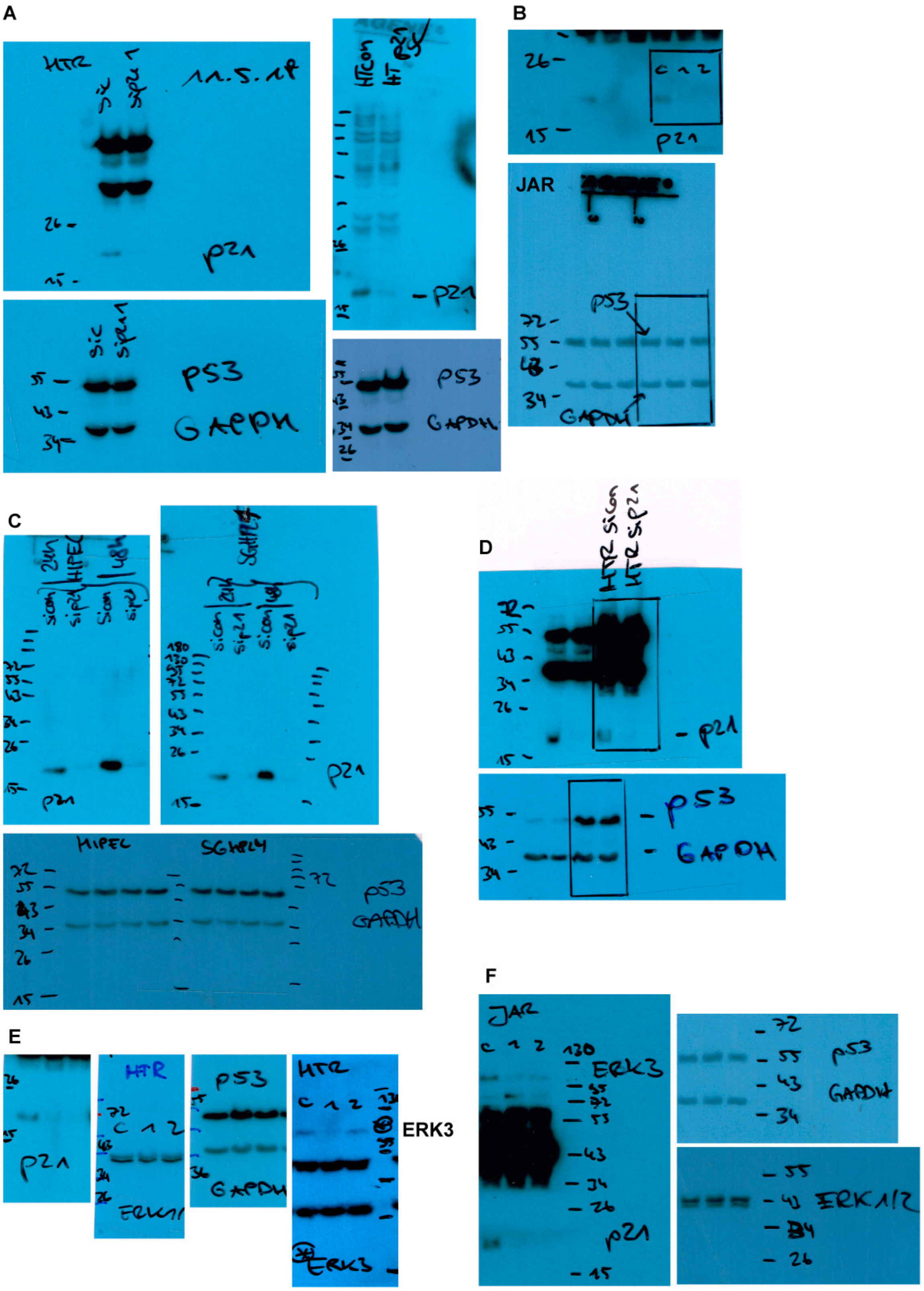

Figure S5. Raw data of Western blots from Figure 4 to Figure 5G. (**A**) Raw data from Figure 4D. (**B**) Raw data from Figure 4G. (**C**) Raw data from Figure 4J,M. (**D**) Raw data from Figure 5A. (**E**) Raw data from Figure 5D. (**F**) Raw data from Figure 5G.

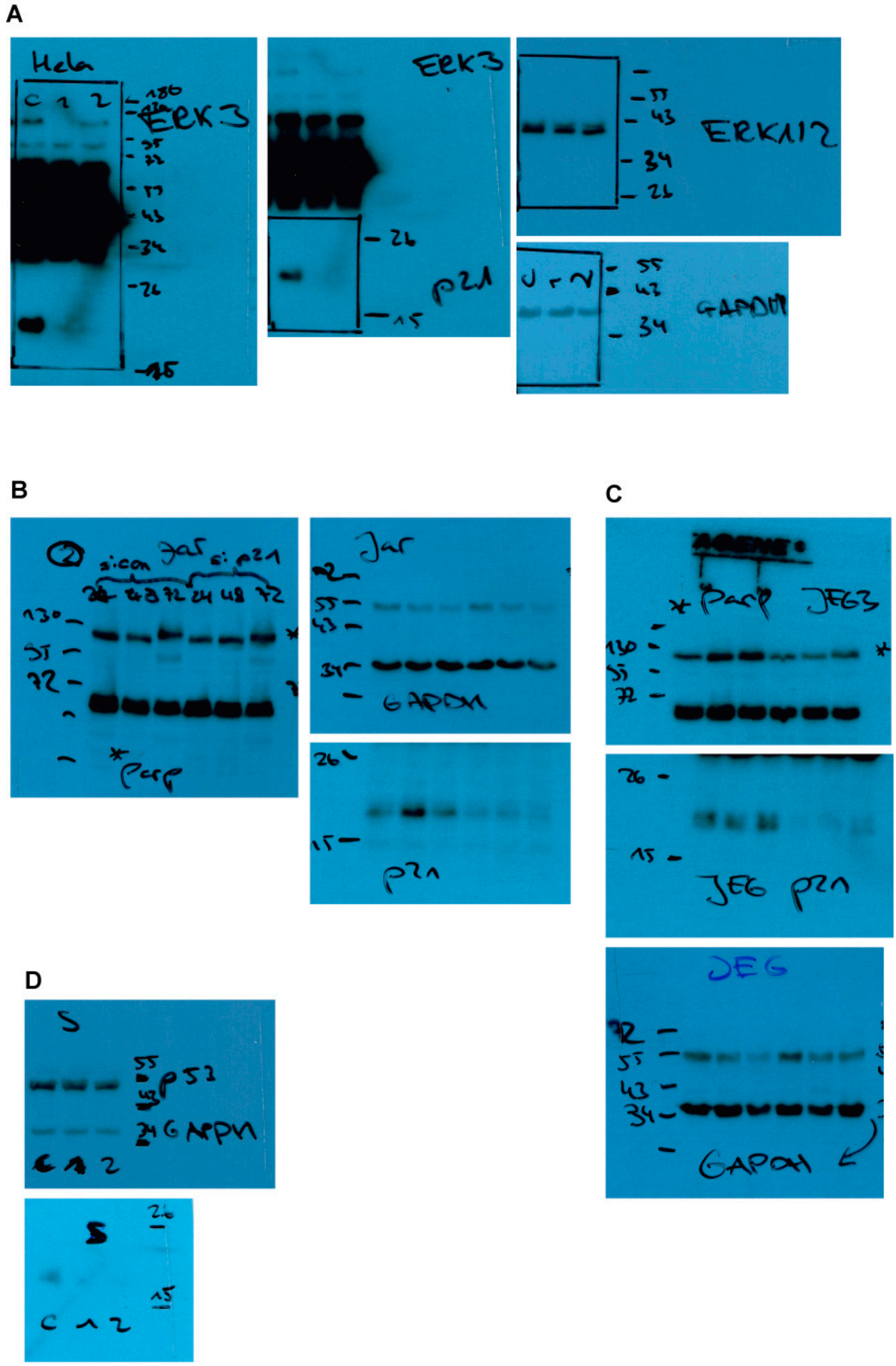

Figure S6. Raw data of Western blots from Figure 5J to Figure S2. (**A**) Raw data from Figure 5J. (**B**) Raw data from Figure S1B. (**C**) Raw data from Figure S1D. (**D**) Raw data from Figure S2A.
